# Long non-coding RNA ZFAS1 promotes pancreatic cancer proliferation and metastasis by sponging miR-497-5p to regulate HMGA2 expression

**DOI:** 10.1038/s41419-021-04123-7

**Published:** 2021-09-22

**Authors:** Min Rao, Song Xu, Yong Zhang, Yifan Liu, Wenkang Luan, Junjing Zhou

**Affiliations:** 1 Hepatobiliary surgery, Affiliated Hospital of Jiangnan University, Wuxi, Jiangsu China; 2 Department of Plastic Surgery, Affiliated People’s Hospital of Jiangsu University, Zhenjiang, Jiangsu China

**Keywords:** Oncogenes, Cell invasion, Long non-coding RNAs

## Abstract

The lncRNA ZFAS1 plays a carcinogenic regulatory role in many human tumours, but it is rarely reported in pancreatic cancer. We identify the role and molecular mechanisms of ZFAS1 in pancreatic cancer. The expression of ZFAS1, miR-497-5p and HMGA2 in pancreatic cancer tissues was detected by qRT-PCR. Pancreatic cancer data in The Cancer Genome Atlas were also included in this study. CCK8, EdU, transwell and scratch wound assays were used to investigate the biological effects of ZFAS1 in pancreatic cancer cells. MS2-RIP, RNA pull-down, RNA-ChIP and luciferase reporter assays were used to clarify the molecular biological mechanisms of ZFAS1 in pancreatic cancer. The role of ZFAS1 in vivo was also confirmed via xenograft experiments. ZFAS1 was overexpressed in pancreatic cancer tissues. ZFAS1 promoted the growth and metastasis of pancreatic cancer cells, and miR-497-5p acted as a tumour suppressor gene in pancreatic cancer by targeting HMGA2. We also demonstrated that ZFAS1 exerts its effects by promoting HMGA2 expression through decoying miR-497-5p. We also found that ZFAS1 promoted the progression of pancreatic cancer in vivo by modulating the miR-497-5p/HMGA2 axis. In conclusion, this study revealed a new role for and the molecular mechanisms of ZFAS1 in pancreatic cancer, identifying ZFAS1 as a novel target for the diagnosis and treatment of pancreatic cancer.

## Introduction

Pancreatic cancer (PC) is the leading cause of cancer-related mortality, with a 5-year survival rate of nearly reached 10% [1, 2]. The characteristics of PC include aggressive local invasion and distant metastasis, and its incidence rate is increasing every year [3]. Despite the continuous advancement of treatment methods including surgery, chemotherapy and radiotherapy, the death rate of PC is still very high [4, 5]. PC progression involves complex changes in multiple genes and pathways [6, 7]. Therefore, the identification of novel biomarkers and therapeutic targets for PC would be of great clinical value.

Long non-coding RNAs (lncRNAs), a class of transcripts over 200 nucleotides with little protein-coding potential, have been proved to modulate various biological processes including apoptosis, invasion, metastasis and angiogenesis in different cancer types [8, 9]. In addition, abnormal expression of lncRNAs has been shown to play a key role in the pathogenesis, development and prognosis of PC [10, 11]. For example, the lncRNA BANCR promotes the tumour carcinogenesis of PC via regulating the miR-195-5p/Wnt/β-Catenin axis [7]. The lncRNA UCA1 is involved in the growth, invasion and migration of PC cells through sponging miR-96 [12].

ZNFX1 antisense RNA1 (ZFAS1), located on chromosome 20q13, is a newly identified tumour-related lncRNA [13, 14]. ZFAS1 was first reported as a tumour suppressor gene in breast cancer [13]. Recent studies have shown that ZFAS1 was overexpressed and promoted the malignant progress of many tumour types such as colorectal cancer, oesophageal squamous cell carcinoma and osteosarcoma [15–17]. However, the specific function and molecular mechanisms of ZFAS1 in PC have not been identified.

In the present study, we found that the expression of ZFAS1 was upregulated in PC and was also critical to PC cell proliferation, invasion and migration. Many studies have found that some lncRNAs act as competitive endogenous RNAs (ceRNAs) to regulate gene expression in many human tumours [9, 18]. ZFAS1 was shown to play the same role in osteosarcoma and papillary thyroid carcinoma [19, 20]. Here, our results show that miR-497-5p suppressed the proliferation and metastasis of PC cells by directly targeting the 3′UTR of HMGA2. ZFAS1 exerts its pro-cancer effects in PC through binding to miR-497-5p to increase the expression of HMGA2. Our findings uncovered that ZFAS1 may be a novel diagnostic indicator and prognostic factor in PC patients.

## Results

### ZFAS1 expression was upregulated in PC tissues and cells and associated with poorer survival of PC patients

**Fig. 1:**
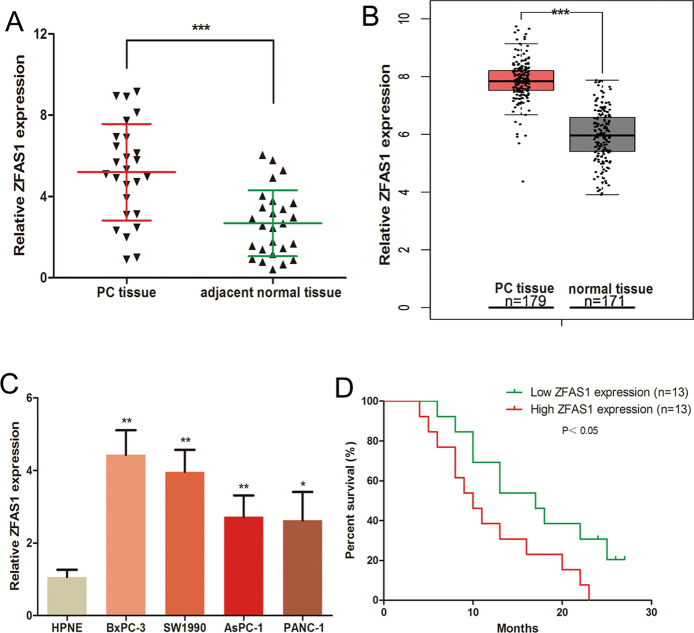
ZFAS1 expression was upregulated in PC tissues and cells and associated with poorer survival of PC patients. **A** The expression of ZFAS1 was measured in 26 PC tissues and adjacent normal tissues. **B** The level of ZFAS1 was analysed in 179 COAD tissues and 171 normal samples by using TCGA database. **C** The ZFAS1 expression in human PC cell lines (BxPC-3, SW1990, AsPC-1 and PANC-1) and human pancreatic ductal cell (HPNE). **D** The overall survival curves of 26 PC tissues. *P < 0.05, **P < 0.01, ***P < 0.001.

We first analysed the level of ZFAS1 in 26 PC tissues and adjacent normal tissues using qRT-PCR. Data showed that the expression of ZFAS1 was higher in PC tissues compared with the adjacent normal tissues (Fig. 1A). To further verify the expression of ZFAS1 in PC, we used GEPIA (http://gepia.cancer-pku.cn/) to analyse the data from The Cancer Genome Atlas (TCGA) database and found the same results (Fig. 1B). Additionally, the expression of ZFAS1 in human PC cell lines (BxPC-3, SW1990, AsPC-1 and PANC-1) was higher than in the human pancreatic ductal cell (HPNE) (Fig. 1C). The Kaplan–Meier survival curve showed that patients with high ZFAS1 expression had a significantly poorer survival rate compared with those with low ZFAS1 expression, based on the PC samples (Fig. 1D).

### Knockdown of ZFAS1 inhibited the proliferation and metastasis of PC cells in vitro

**Fig. 2:**
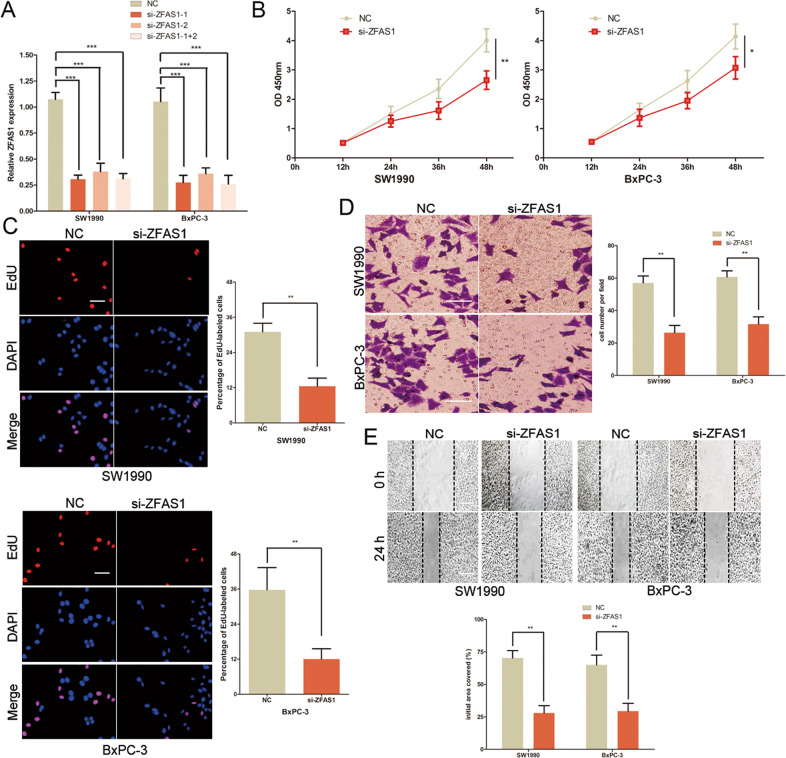
Knockdown of ZFAS1 inhibited the proliferation and metastasis of PC cells in vitro. **A** Transfection efficiency of ZFAS1 siRNA was determined by using qRT-PCR. **B** The proliferative ability of SW1990 and BxPC-3 cells was determined by CCK8 assay. **C** The DNA synthesis of SW1990 and BxPC-3 cells grown was measured by EdU assay. Scale bar, 100 μm. **D** The invasive capacity of SW1990 and BxPC-3 cells was assessed by the transwell assay. Scale bar, 100 μm. **E** The effect of ZFAS1 siRNA on the migratory ability of SW1990 and BxPC-3 cells was assessed by the scratch wound assay. Scale bar, 200 μm.*P < 0.05, **P < 0.01, ***P < 0.001.

To investigate the biological role of ZFAS1 in PC cells, the siRNA for ZFAS1 was transfected into SW1990 and BxPC-3 cells (Fig. 2A). CCK-8 assays showed that the proliferation of SW1990 and BxPC-3 cells was repressed after knockdown of ZFAS1 (Fig. 2B). EdU assay also revealed that the number of EdU-positive cells were significantly decreased in PC cells after transfection with ZFAS1 siRNA (Fig. 2C). Furthermore, transwell and wound healing assays were used to detect the invasion and migration of PC cells. The migration and invasion of ZFAS1 siRNA-transfected SW1990 and BxPC-3 cells were significantly inhibited compared with the control group (Fig. 2D, E). These results collectively showed that ZFAS1 promoted the proliferation and metastasis of PC cells.

### ZFAS1 acted as a miR-497-5p sponge in PC

**Fig. 3:**
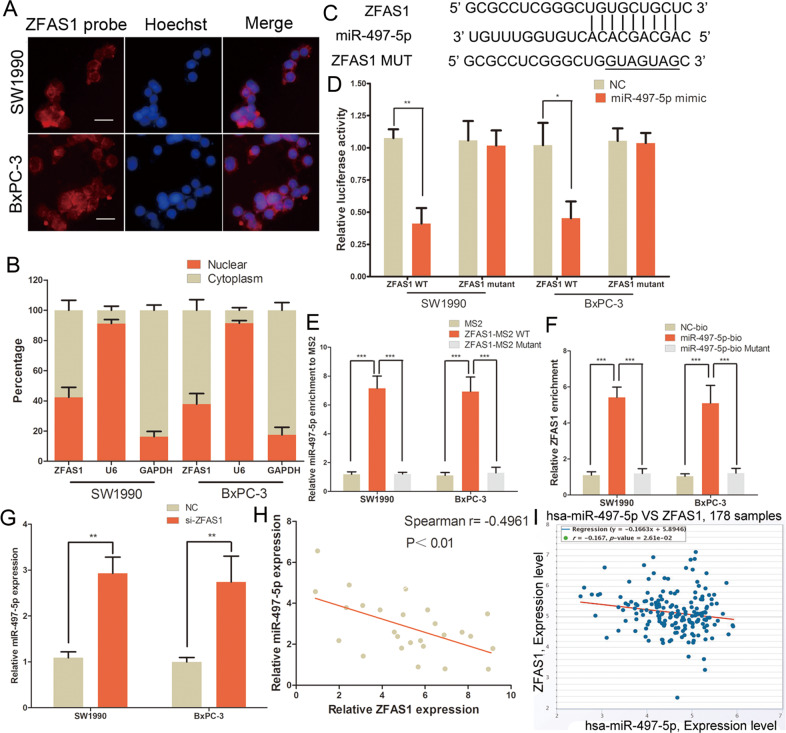
ZFAS1 acted as a miR-497-5p sponge in PC. **A** FISH showed that ZFAS1 was mainly distributed in both cytoplasm and nucleus in PC cells. Scale bar, 25 μm. **B** qRT-PCR detected the ZFAS1 level in cytoplasm and nuclear. **C** The binding sites of miR-497-5p on the ZFAS1. **D** Luciferase activity of PC cells transfected with ZFAS1-WT or ZFAS1-MUT reporter together with miR-497-5p or NC. **E** MS2-RIP followed by miRNA qRT-PCR to detect endogenous miR-497-5p associated with the MS2-tagged ZFAS1 in PC cells. **F** PC cells were transfected with biotin-labelled miR-497-5p, and assayed by biotin-pull-down. ZFAS1 levels were analysed by qRT-PCR. **G** The expression of miR-497-5p in PC cells following transfection with ZFAS1 siRNA or NC. **H** The correlation of miR-497-5p and ZFAS1 expression in 26 PC tissues was negative. **I** TCGA dataset revealed a negative correlation between miR-497-5p and ZFAS1 expression in PC tissues. *P < 0.05, **P < 0.01, ***P < 0.001.

Some lncRNAs have been reported to play the role of ceRNAs to mediate miRNAs in various tumours [9, 21]. We first found that ZFAS1 was localised in both the cytoplasm and nuclei using ZFAS1-FISH assay and qRT-PCR on PC cell fragments (Fig. 3A, B). LncRNASNP2 (http://bioinfo.life.hust.edu.cn/lncRNASNP#!/) and Starbase 3.0 (http://starbase.sysu.edu.cn) were then used to identify miRNAs that may bind to ZFAS1, and we found that miR-497-5p has potential binding sites (Fig. 3C). Luciferase reporter assay in PC cells demonstrated that miR-497-5p mimics caused inhibition of luciferase activity in the ZFAS1 wild-type vector but not the mutant vector (Fig. 3D). MS2-RIP and RNA pull-down assays were used to further verify the direct binding of miR-497-5p and ZFAS1 in PC. Compared with the empty plasmid and mutant groups, the MS2-tagged wild-type ZFAS1 vector was enriched for miR-497-5p in PC cells (Fig. 3E). Additionally, ZFAS1 was pulled down using biotinylated miR-497-5p in PC cells where no significant change was found in the NC and mutation group (Fig. 3F). Furthermore, miR-497-5p expression was suppressed by ZFAS1 siRNA in SW1990 and BxPC-3 cells (Fig. 3G). We also found that ZFAS1 and miR-497-5p expression were inversely correlated in PC tissues, based on our samples and the TCGA database (Fig. 3H, I). These results demonstrated that miR-497-5p and ZFAS1 directly bind in PC.

### ZFAS1 promoted HMGA2 expression through sponging miR-497-5p in PC

**Fig. 4:**
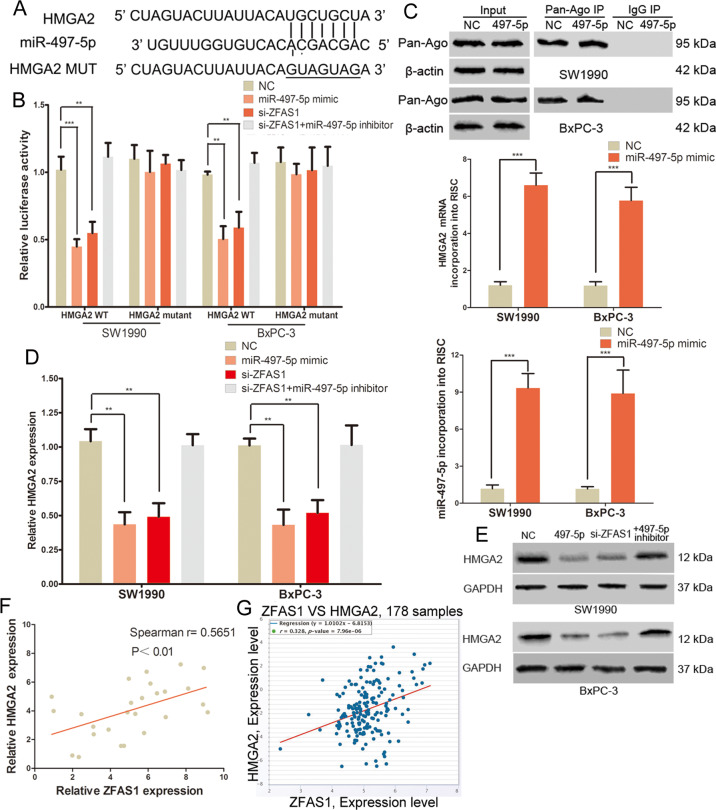
ZFAS1 promoted HMGA2 expression through sponging miR-497-5p in PC. **A** The binding sites of miR-497-5p within the 3′-UTR of HMGA2. **B** Luciferase activity of PC cells transfected with HMGA2-WT or HMGA2-MUT reporter. **C** Immunoprecipitation of the Ago2/RISC using the Pan-Ago2 antibody in PC cells overexpressing miR-497-5p. IgG was used as a negative control, β-actin was used as an internal control. qRT-PCR analysis of miR-497-5p and HMGA2 incorporated into RISC in overexpressing miR-497-5p PC cells. **D** The expression of HMGA2 mRNA in PC cells transfected with miR-497-5p mimic, ZFAS1 siRNA or ZFAS1 siRNA plus miR-497-5p inhibitor. **E** Western blots identified HMGA2 protein expression changes, GAPDH was used as a control. **F** The correlation of HMGA2 and ZFAS1 expression in 26 PC tissues was positive. **G** TCGA dataset revealed a significant positive correlation between HMGA2 and ZFAS1 in PC tissues. *P < 0.05, **P < 0.01, ***P < 0.001.

Bioinformatics software (TargetScan, miRDIP, miRDB and miRPathDB) was used to predict target genes of miR-497-5p. Results identified HMGA2 as a potential target (Fig. 4A). The 3′-UTR of HMGA2 has the same miR-497-5p binding sites as miR-497-5p combined with ZFAS1 (Fig. 4A). Luciferase reporter assay showed that the luciferase activity of the wild-type HMGA2 vector was decreased in the miR-497-5p mimic-transfected group (Fig. 4B). We detected the abundance of HMGA2 mRNA in Ago2/RNA induced silencing complex (RISC) after up-regulation of miR-497-5p, and found that the levels of HMGA2 and miR-497-5p were enriched in RISC in PC cells with miR-497-5p overexpression (Fig. 4C). Furthermore, HMGA2 mRNA and protein levels were suppressed by the miR-497-5p mimic (Fig. 4D, E). Moreover, the luciferase activity of the wild-type HMGA2 vector was inhibited upon knockdown of ZFAS1 in PC cells (Fig. 4B). ZFAS1 siRNA suppressed the mRNA and protein expression of HMGA2 in SW1990 and BxPC-3 cells (Fig. 4D, E). These effects of ZFAS1 were reversed by the miR-497-5p inhibitor (Fig. 4B, D, E). The PC tissue samples and TCGA datasets also showed that ZFAS1 and HMGA2 mRNA are positively correlated in PC (Fig. 4F, G). These results suggested that ZFAS1 upregulated HMGA2 expression by decoying miR-497-5p in PC.

### ZFAS1 regulated the growth and metastasis of PC cells through the miR-497-5p/HMGA2 axis

**Fig. 5:**
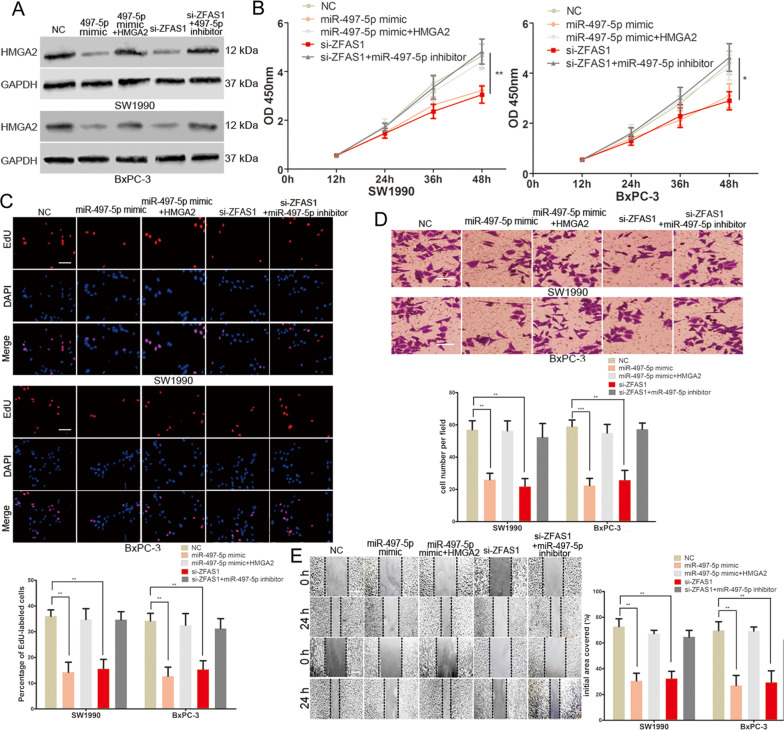
ZFAS1 regulated the growth and metastasis of PC cells through the miR-497-5p/HMGA2 axis. **A** Western blots identified HMGA2 protein expression changes in transfected PC cells, GAPDH was used as a control. **B** The proliferative ability of PC cells was determined by CCK8 assay. **C** The DNA synthesis of PC cells grown was measured by EdU assay. Scale bar, 100 μm. **D** The invasive capacity of SW1990 and BxPC-3 cells was assessed by the transwell assay. Scale bar, 100 μm. **E** The migratory ability of SW1990 and BxPC-3 cells was assessed by the scratch wound assay. Scale bar, 200 μm. *P < 0.05, **P < 0.01, ***P < 0.001.

We further explored whether ZFAS1 plays a carcinogenic role in PC through the miR-497-5p/HMGA2 axis. We transfected ZFAS1 siRNA, miR-497-5p mimic, miR-497-5p mimic plus the HMGA2 plasmid and ZFAS1 siRNA plus the miR-497-5p inhibitor into PC cells and measured HMGA2 protein levels in different groups via western blotting (Fig. 5A). miR-497-5p inhibited the proliferation, invasion and migration of PC cells where HMGA2 reversed the role of miR-497-5p (Fig. 5B–E). These results demonstrated that miR-497-5p plays a tumour suppressor role by targeting HMGA2 in PC. The inhibitory effect of ZFAS1 siRNA on the HMGA2 levels was reversed by the miR-497-5p inhibitor (Fig. 5A). Moreover, CCK-8, EdU, transwell and wound healing assays showed that the effect of ZFAS1 siRNA on the growth and metastasis of PC cells was abolished when co-transfected with the miR-497-5p inhibitor (Fig. 5B–E). In summary, these data indicated that ZFAS1 exerts its role in PC by sponging miR-497-5p to upregulate HMGA2 expression.

### ZFAS1 exerts its oncogenic activity through regulating the miR-497-5p/HMGA2 axis in vivo

**Fig. 6:**
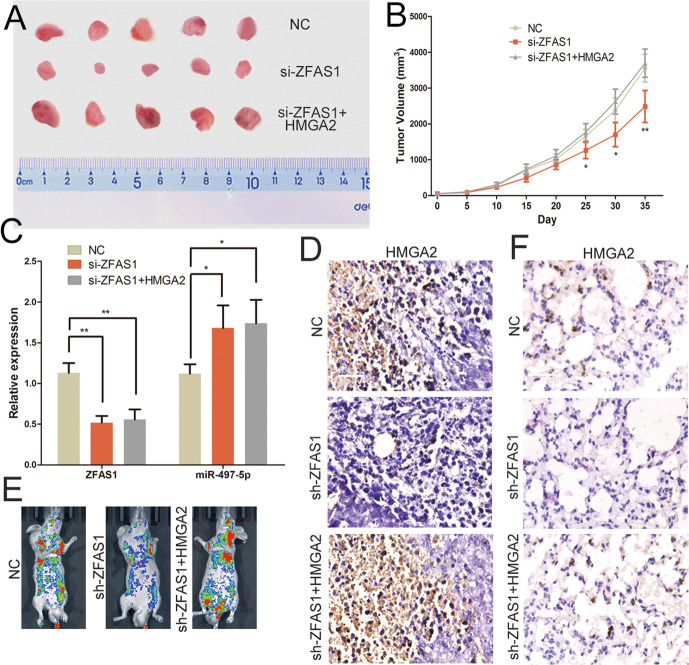
ZFAS1 exerts its oncogenic activity through regulating the miR-497-5p/HMGA2 axis in vivo. **A** The excision tumour in nude mice of PC xenograft model. **B** Differences in tumour volume among groups. **C** PCR identified ZFAS1 and miR-497-5p expression changes. **D** The expression of HMGA2 was examined by immunohistochemical staining of sections from the xenograft model. Scale bar, 25 μm. **E** Representative bioluminescence images of mice after tail vein injection of stably expressing sh-ZFAS1 or sh-ZFAS1 plus HMGA2 plasmid SW1990 cells. **F** The expression of HMGA2 were detected by immunohistochemistry of sections from the metastatic lung nodules. Scale bar, 25 μm. *P < 0.05, **P < 0.01, ***P < 0.001.

To explore the role of ZFAS1 in vivo, the PC xenograft model was established. After 35 days, all tumours were removed from the nude mice (Fig. 6A). Compared with the control group, tumour growth was inhibited in the ZFAS1 knockdown group from 25 to 35 days, and this inhibitory effect was reversed by HMGA2 plasmid (Fig. 6B). The levels of ZFAS1, miR-497-5p and HMGA2 in the excised tumour sections were measured after 35 days. miR-497-5p expression was upregulated and HMGA2 expression was downregulated in the ZFAS1 knockdown group, the effect of ZFAS1 siRNA on the miR-497-5p and HMGA2 levels was abolished by HMGA2 plasmid (Fig. 6C, D). In order to study the effect of ZFAS1 on the metastasis of PC cells in vivo, SW1990 cells that stably expressing shRNA of ZFAS1 was injected into nude mice via tail vein. The knockdown group of ZFAS1 showed lower in vivo colonisation levels compared with the control group (Fig. 6E). HMGA2 was reduced in ZFAS1 knockdown group when we analysed the sections of metastatic pulmonary nodules (Fig. 6F). HMGA2 plasmid also reversed the effect of ZFAS1 shRNA on metastasis of PC cells in vivo (Fig. 6E, F). These results demonstrated that ZFAS1 promoted the progression of PC in vivo through modulating the miR-497-5p/HMGA2 axis.

## Discussion

LncRNAs are transcripts with a length <200 nucleotides and weak protein-coding capacity [22, 23]. A large number of studies have shown that some lncRNAs are abnormally expressed and play a crucial role in the occurrence and development of many human tumours [9, 12, 18]. ZFAS1, mapped to chromosome 20q13, is an antisense transcript of the gene zinc finger NFX1-type containing 1 (ZNFX1) at the 5′-end [13]. ZFAS1 was first identified as a tumour suppressor gene in breast cancer [13]. With the development of further research, many studies reported that ZFAS1 is overexpressed in and promotes the malignant progression of many types of human tumours [24, 25]. However, the function and mechanisms of ZFAS1 in pancreatic cancer have not been reported.

In the present report, we found that ZFAS1 is highly expressed in pancreatic cancer, and a high level of ZFAS1 is a risk factor for a poor prognosis in patients with pancreatic cancer. ZFAS1 enhanced the proliferation and metastasis of PC cells. A large number of studies have shown that some special lncRNAs play the role of ceRNAs in many human tumours [26, 27]. ceRNAs regulate the level of oncogenes and tumour suppressor genes by sponging miRNAs to reduce the binding between miRNAs and target genes [28]. Similarly, it has been reported that ZFAS1 participates in the progression of colorectal cancer through the miR-150-5p/VEGFA axis [16]. Exosomal ZFAS1 affects the growth and apoptosis of oesophageal squamous cell carcinoma cells through sponging miR-124 to regulate STAT3 expression [15]. Here, we further investigated whether ZFAS1 has the same effect in PC. We found that ZFAS1, miR-497-5p and HMGA2 were potentially correlated with ceRNAs in PC using bioinformatics software. MS2-RIP, RNA pull-down and Luciferase reporter assays confirmed the direct binding of ZFAS1 and miR-497-5p in PC. We predicted and confirmed that HMGA2 is a target gene of miR-497-5p. Moreover, knockdown of ZFAS1 decreased HMGA2 levels and the luciferase activity of a wild-type HMGA2 luciferase reporter plasmid, which was abolished using miR-497-5p inhibitor. All the results showed that ZFAS1 induced the increased expression of HMGA2 in PC by sponging miR-497-5p.

miR-497-5p, a tumour suppressor, is downregulated in many cancers and inhibits the progression of malignant tumours [29, 30]. HMGA2 was identified as a oncogene in pancreatic cancer [31]. HMGA2 is associated with tumour grade, growth, metastasis, poor prognosis and epithelial mesenchymal transition of PC [31, 32]. It has also been reported that HMGA2 plays an important role in the formation of chemoresistance in PC [33]. Here, we confirmed that miR-497-5p plays a role in inhibiting the biological phenotype of PC cells by targeting HMGA2. In addition, we further found that the effect of ZFAS1 siRNA on the proliferation and metastasis of PC cells was reversed by a miR-497-5p inhibitor. We finally confirmed that ZFAS1 promoted the growth and metastasis of PC through modulating the miR-497-5p/HMGA2 axis in vivo. This study demonstrated that ZFAS1 exerts its effects on PC by sponging miR-497-5p to upregulate HMGA2 levels.

Taken together, these results demonstrated that ZFAS1 is involved in the malignant progression of PC. ZFAS1 decreased binding between miR-497-5p and HMGA2 by decoying miR-497-5p, which led to the up-regulation of HMGA2. ZFAS1 promoted the growth and metastasis of PC by modulating the miR-497-5p/HMGA2 axis. Understanding the mechanisms of the ZFAS/miR-497-5p/HMGA2 ceRNA network in PC will be helpful for identifying new biomarkers or therapeutic targets in patients with pancreatic cancer.

## Methods

### Human tissues

Pancreatic cancer tissues and adjacent normal tissues were collected from patients with pancreatic cancer who did not receive chemoradiotherapy before surgery in the general surgery department of Affiliated Hospital of Jiangnan University. The pathological diagnosis was independently performed by two pathologists. The study was approved by the ethics committee of the Affiliated Hospital of Jiangnan University. The public database of The Cancer Genome Atlas (TCGA) was also used to analyse the expression of related molecules in pancreatic cancer.

### Cell lines, plasmids, oligonucleotides and transfection

Four human PC cell lines (BxPC-3, SW1990, AsPC-1 and PANC-1) and one human pancreatic ductal cell (HPNE) were obtained from the American Type Culture Collection (ATCC, USA). Dulbecco’s modified Eagle’s medium (DMEM; Gibco, USA) containing 10% fetal bovine serum (Invitrogen, USA) was used to culture the cell lines. These cell lines were grown in a humidified incubator with an atmosphere of 5% CO^2^ at 37 °C. The miR-497-5p mimic, miR-497-5p inhibitor and the small interfering RNA (siRNA) of ZFAS1 were chemically synthesised by GenePharma (Shanghai, China). The sequences were as follows: ZFAS1-small interfering RNA-1 (si-ZFAS1-1), 5′-AGCCATCTTTGGTTATATAAGGGAGGTTC-3′; ZFAS1-small interfering RNA-2 (si-ZFAS1-2), 5′-CCACGTGCAGACATCTACAACCTTCGATC-3′. Full-length HMGA2 was amplified and inserted into a pcDNA3.1 vector (Invitrogen, USA) to construct the HMGA2 plasmid. The corresponding plasmids and oligonucleotides were transfected into PC cells using lipofectamine 3000 (Invitrogen, USA). The short hairpin RNA (shRNA) of ZFAS1 were also synthesised by GenePharma (Shanghai, China). The shRNA were inserted into the lentivirus vector, and the stably expressing shRNA cells were constructed by infecting cells with the lentivirus.

### Western blotting

Protein was extracted using RIPA buffer (KenGEN, China), and the concentration of the extracted protein was determined by a BCA Protein Assay Kit (Beyotime, China). Western blotting was carried out as reported previously [34]. An HMGA2 antibody (Abcam, 1:1000, UK) was used to detect the protein level of HMGA2, and GAPDH (1:2500, Abcam, UK) was used for normalisation.

### Quantitative RT-PCR

RNA was extracted from cells and tissues using TRIzol (Invitrogen, USA), and reverse transcription was performed using Fermentas and microRNA reverse transcription kits (Applied Biosystems, CA). The ABI StepOnePlus System (Applied Biosystems, CA) was used for the amplification reaction using the recommended reaction conditions. The miR-497-5p primer was chemically synthesised by RiboBio (Guangzhou, China). The following primers were used: ZFAS1 forward 5′-GCTATTGTCCTGCCCGTTAG-3′ and ZFAS1 reverse 5′-TCGTCAGGAGATCGAAGGTT-3′; HMGA2 forward 5′-ACCCAGGGGAAGACCCAAA-3′ and HMGA2 reverse 5′-CCTCTTGGCCGTTTTTCTCCA-3′. U6 and GAPDH were used for normalisation, and 2^–ΔΔCt^ was used to calculate the relative expression.

### Cell proliferation assay

Cell proliferation was measured using the cell counting kit-8 (CCK-8, Beyotime, Shanghai, China). Approximately 5000 PC cells were inoculated into 96-well plates, and 100 μl culture medium containing 10% CCK8 was added to each well. The plates were incubated for 12, 24, 36, and 48 h. A microplate reader (Multiscan FC, Thermo Scientific) was used to detect the absorbance at 450 nm optical density. DNA synthesis of growing PC cells was measured via EdU assay using the EdU imaging kit (Life Technologies, USA), and the assay steps were carried out according to the manufacturer’s instructions. Immunohistochemical staining was observed with a Leicadmi 3000B microscope, and the number of positive cells was counted.

### Cell metastasis assay

The invasion of PC cells was detected by transwell assay. The transfected PC cells were suspended in serum-free DMEM and placed in the upper layer of the Matrigel covered chamber (BD Biosciences, USA). Medium containing 10% fetal bovine serum was added to the lower layer as a chemical attractant. The invasive cells were fixed, stained and photographed after 24 h. The migration of PC cells was detected by scratch wound assay. We seeded the transfected PC cells into a six-well plate and scratched the cell layer with a 200-μl pipette to form a wound space. We used the medium containing 1% serum to eliminate or reduce the effect of proliferation. The wound width of the cell layer was recorded at 0 and 24 h.

### Fluorescence in situ hybridization

According to the previous research steps, Fluorescence in situ hybridization (FISH) was carried out using RiboTM Fluorescent In Situ Hybridization Kit (ribobio, Guangzhou, China). We commissioned RiboBio (Guangzhou, China) to synthesise the probe of ZFAS1, and cell nucleus were stained with Hoechst. We used confocal microscopy to obtain representative photographs.

### Luciferase reporter assay

We inserted fragments of the HMGA2 3′-UTR and ZFAS1 that contained the miR-497-5p binding site into a REPORT plasmid and mutated the binding site to construct the mutant plasmid as the control. miR-497-5p mimics, negative control and related reporter plasmids were transfected into PC cells. The corresponding luciferase activity was determined by a Dual-Luciferase Reporter Assay System (Promega, USA) after 24 h of transfection.

### Isolation and detection of RISC-associated RNA

We transfected miR-497-5p mimics into PC cells and then fixed the cells with 1% paraformaldehyde. The cells were incubated with Dynabeads Protein A (Invitrogen, USA) with IgG or anti-Pan-Ago, and clone 2A8 antibodies (Millipore, USA) after lysing with NETN buffer. The immunoprecipitated RNA was released after digestion with Proteinase K (Invitrogen, USA). RNA was extracted and purified and treated with DNase I. QRT-PCR was used to detect the level of miR-497-5p and HMGA2 in the immunoprecipitated RNA.

### RNA pull-down assay

We synthesised biotinylated miR-497-5p through GenePharma (Shanghai, China), and mutant and NC were used as the control. The synthesised oligonucleotides were then transfected into PC cells, and M-280 streptavidin magnetic beads (Invitrogen, USA) were incubated with the cell lysates. We then extracted the bound RNA and detected the content of ZFAS1 by qRT-PCR.

### MS2-RIP assay

To identify miRNA binding with ZFAS1, maltose-binding protein (MBP)-affinity purification technology was used. MS2-MBP was expressed and purified from *E. coli*, and the sequence downstream of ZFAS1 was inserted with three bacteriophage MS2 coat protein-binding sites using a Site-Directed Mutagenesis Kit (Invitrogen, USA). MS2 labelled ZFAS1 was transfected into PC cells. The corresponding cells were subjected to RIP analysis after 48 h, and the miR-497-5p level was measured using qRT-PCR.

### Xenograft tumour assay, in vivo metastasis assay and immunohistochemistry staining

Nude mice were injected subcutaneously with SW1990 cells to form the xenograft tumours. After the tumour volume reached 50 mm^3^, mice were divided into three groups. The tumour volume calculation formula was as follows: length × width^2^ × 0.5. ZFAS1 siRNA, ZFAS1 siRNA plus HMGA2 plasmid or NC was injected into the interior of the tumours every 5 days for 35 days, and the tumour volume was calculated each time. The tumour tissue was dissected and photographed after 35 days. SW1990 stably expressing sh-ZFAS1 or sh-ZFAS1 plus HMGA2 plasmid were injected into the tail vein of mice. Sterile D-Luciferin firefly potassium salt (Beyotime, China) were injected into nude mice. In vivo imaging was performed by using PerkinElmer IVIS Spectrum (Xenogen, CA) and quantified by using Living Image software (Xenogen, CA). After 21 days, the lung was dissected. The subcutaneous tumour tissue and metastatic pulmonary nodules were sectioned, and immunohistochemical staining was carried out using an HMGA2 antibody (Abcam, UK). The experimental steps were performed according to the methods described previously [35]. This research was endorsed by the Research Ethics Committee of the Affiliated Hospital of Jiangnan University.

### Statistical analysis

The data (mean ± SD) were evaluated by SPSS 13.0. The statistical significance between the data was evaluated by *t* test. We used MATLAB to analyse the spearman correlation and Kaplan–Meier survival curve to evaluate the relationship between ZFAS1 level and PC patient survival. PC tissues were divided into two groups according to the ZFAS1 level, and a log rank test was used to test the statistical difference between the curves. The corresponding statistical pictures were drawn with GraphPad Prism 5. A *P* value < 0.05 was considered statistically significant.
